# Impact of Postoperative Infection on Lower Limb Function After Surgery for Malignant Bone and Soft Tissue Tumors: Data from a Nationwide Registry in Japan

**DOI:** 10.3390/curroncol32080454

**Published:** 2025-08-13

**Authors:** Takeshi Morii, Kenji Sato, Koichi Ogura, Tomohiro Shinozaki, Akira Kawai

**Affiliations:** 1 Department of Orthopaedic Surgery, School of Medicine, Kyorin University, 6-20-2 Shinkawa, Mitaka 181-8611, Tokyo, Japan; 2 Department of Musculoskeletal Oncology and Rehabilitation Medicine, National Cancer Center Hospital, 5-1-1 Tsukiji, Chuo-ku 104-0045, Tokyo, Japan; kenji@med.teikyo-u.ac.jp (K.S.); koogura@ncc.go.jp (K.O.); akawai@ncc.go.jp (A.K.); 3 Interfaculty Initiative in Information Studies, The University of Tokyo, 7-3-1 Hongo, Bunkyo-ku 113-0033, Tokyo, Japan; shinozaki.tomohiro@iii.u-tokyo.ac.jp

**Keywords:** malignant bone and soft tissue tumor, limb function, nationwide registry, postoperative infection

## Abstract

Background: Due to the scarcity of cases, adjustments for confounding factors in analyses of the effects of postoperative infections on limb function after surgery for malignant bone and soft tissue tumors were insufficient in previous studies. One solution is to use big data from a nationwide registry. Methods: Data from the Bone and Soft Tissue Tumor Registry in Japan were used to examine the impact of postoperative infections on limb function after surgery for malignant bone and soft tissue tumors in the lower extremities. Limb function was evaluated using the Musculoskeletal Tumor Society (MSTS) score. Results: A total of 1099 soft tissue tumors and 410 bone tumor cases were included. Propensity score matching (PSM) was performed using significant factors in the propensity score logistic regression. After PSM, only “function” and “emotional acceptance” scores worsened in infection cases in the bone tumor group, while total MSTS scores remained unaffected in both the bone and soft tissue tumor groups. No subcategory scores worsened in the soft tissue tumor group. Conclusions: Our findings suggest that postoperative infections might not affect function in cases of soft tissue tumors and have only a limited impact in cases of bone tumors.

## Simple Summary

Surgery for cancer in the bones and soft tissues of the legs can sometimes lead to infections. These infections might affect how well patients can use their legs in daily life. However, past studies had too few cases to clearly understand this effect. In this study, we used a large national database in Japan to examine whether postoperative infections influence patients’ ability to return to normal life after surgery. We analyzed over 1500 cases and compared patients with and without infections in a balanced way. Although patients who had infections tended to have worse use of their legs, our analysis—taking other health and treatment differences into account—suggests that infection itself may have only a limited direct effect on leg function.

## 1. Introduction

While limb salvage surgery for malignant bone and soft tissue tumors with proper margins provides acceptable oncological outcomes, it is associated with a considerably high incidence of complications, which still poses a problem. The lack of sufficient soft tissue due to wide resection, fibrosis due to perioperative radiotherapy, immunosuppression due to perioperative chemotherapy, and reconstruction by megaprosthesis all negatively impact infection prevention. Indeed, surgical site infections are significantly more common after tumor surgery than after conventional orthopedic surgery [1].

Postoperative complications, including infections, have been reported to have devastating effects on several areas, including prolonged hospital stay [2], delayed start of adjuvant radiation therapy [2], and increased cost [3]. In addition, strong relationships between infection/wound trouble and reduced quality of life (QOL) and limb function have been sporadically reported [4,5,6,7,8]. Because risk factors for worse limb function, such as greater age, higher body mass index (BMI), radiotherapy, plastic surgery reconstruction, female sex, large size, and nerve resection, were also identified as candidate risk factors for infection [1,5,6,9,10,11], adjusting for these variables using multivariable-adjusted regression models or propensity score adjustment is needed to demonstrate unconfounded associations. The fundamental challenge in such studies lies in the difficulty of accumulating a sufficient number of cases due to the rarity of malignant bone and soft tissue tumors, which are representative of rare cancers. To date, an ideal cohort suitable for such an analysis has not been achieved.

In this study, we use a nationwide registration-based approach to accumulate sufficient data to analyze the effect of postoperative infections on lower limb function. The Bone and Soft Tissue Tumor (BSTT) Registry was launched in Japan in the 1950s by the Japanese Orthopaedic Association (JOA) and promoted by the National Cancer Center. All JOA-certified hospitals for musculoskeletal oncology (*N* = 89) must participate in this registry [1]. In 2006, the database was renovated as a digital chart-based system, and in 2014, it became available for clinical research.

## 2. Materials and Methods

**Figure 1:**
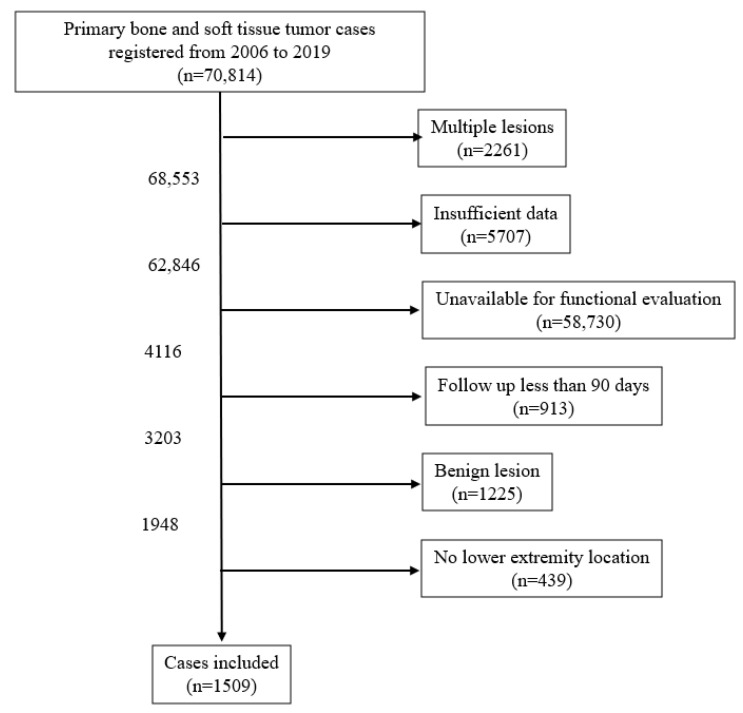
Flowchart of the inclusion of cases from the overall BSTT Registry data.

This retrospective study used nationwide registry data extracted from the BSTT Registry, which includes almost all musculoskeletal malignant tumor cases in Japan. From 2006 to 2019, 70,814 primary bone and soft tissue tumor cases were registered. The case extraction process is shown in Figure 1. The exclusion criteria were multiple lesions, insufficient data, lack of functional evaluation, follow-up of <90 days, benign lesions, and locations other than in the lower extremities. The lower limit of the follow-up period was based on the diagnostic criteria for surgical site infection by the Centers for Disease Control and Prevention (CDC) in 2017 [12]. Insufficient data were defined as cases in which the independent variables investigated in this study had missing values in the database used. Ultimately, 1509 cases were included in the analysis. Limb function was evaluated using the Musculoskeletal Tumor Society (MSTS) score [13]. The MSTS score for lower limbs consists of six scoring items: “pain,” “function,” “emotional acceptance,” “external support,” “functional independence,” and “gait,” which were measured at the final follow-up. Postoperative infection was defined as cases diagnosed by the surgeons as presenting infections that needed surgical intervention to control. In the BSTT Registry, additional surgical interventions for managing postoperative events, including infections, must be registered for each case. We extracted the cases with surgical interventions for infection control using this scheme.

Before comparing functional outcomes between cases with and without postoperative infections, propensity score matching (PSM) was used to align their backgrounds and characteristics, including age, sex, tumor origin (bone or soft tissue), pelvic bone location, tumor diameter, tumor grade, application of myocutaneous flap, reconstruction by prosthesis, perioperative chemotherapy, and radiotherapy. Propensity scores were estimated using a logistic regression model that included these variables, and the caliper for matching was defined as 0.2 of the standard deviation of the logit of the propensity score. The distribution of MSTS scores was compared between cases with and without postoperative infections using Student’s *t*-tests. The results are presented as the mean ± standard deviation in each group and the differences with a 95% confidence interval between groups.

Statistical analyses were performed using the JMP software (version 13.0.0; SAS Institute Inc., Cary, NC, USA).

## 3. Results

The patients’ demographic and tumor characteristics are summarized in Table 1. There were 1099 soft tissue tumor and 410 bone tumor cases, of which 38 (3.4%) soft tissue tumor cases and 43 (10.5%) bone tumor cases were diagnosed with postoperative infections.

Table 2 shows the estimated odds ratios in the propensity score logistic regression model for postoperative infection. Tumor grade and reconstruction by prosthesis in soft tissue tumors, and pelvic bone location and application of myocutaneous flap in bone tumors were identified as significant factors. In the bone tumor group, infection cases tended to have a longer follow-up period, so this was also included as an adjustment factor. PSM was performed using these factors, and patients’ characteristics after PSM are shown in Table 3.

Finally, we confirmed the impact of postoperative infections on MSTS scores using this data set. As shown in Table 4, in soft tissue tumors, the MSTS score was suggested to be associated with infection before propensity score matching (PSM), but this association was not observed after PSM. Similarly, in bone tumors, although the MSTS score appeared to be associated with infection before PSM, after matching, statistically significant functional decline in infected cases was observed only in the domains of “function” and “emotional acceptance”.

## 4. Discussion

The practical management modalities of surgical site infections after bone and soft tissue tumor surgeries include debridement, antibiotics, and implant retention (DAIR) and one- or two-stage revision [14]. In order to control infection by surgical intervention, sufficient debridement of the infected area is inevitable, resulting in a lack of viable soft tissue/muscle and limb weakness. Long-term fixation of the affected limb, which is sometimes used after two-stage revision, and fibrosis due to inflammatory changes result in limb stiffness. Theoretically, all these local events might lead to functional loss of the limb. However, in the present analysis, before applying PSM, infection and the associated surgical interventions seemed to affect limb function, but after applying PSM, the impact of infection on lower limb function was not clearly demonstrated in cases with soft tissue tumors. In bone tumors, a possible association was suggested only in specific items—function and emotional acceptance—with postoperative infections requiring surgical intervention. The loss of statistical significance after propensity score matching implies that the observed differences in MSTS scores were at least partly attributable to baseline imbalances, and not solely due to the presence of infection. Specifically, in the soft tissue tumor group, high tumor grade and the use of endoprosthetic reconstruction may have contributed to functional impairment. In the bone tumor group, pelvic tumor location and muscle flap reconstruction were more common in infection cases and are known to negatively affect functional outcomes. Moreover, infection cases tended to have longer follow-up periods, potentially increasing the chance of infection detection and subsequent functional decline.

The effects of wound complications on the clinical outcomes of malignant bone or soft tissue tumor surgery, including function and QOL, have been sporadically studied. It has been reported that wound trouble, including infection, can result in loss of function or QOL. For example, wound complications, defined as any surgical complication requiring an interventional procedure, any wound complication requiring deep packing for longer than four weeks, or any neurologic complication involving new motor deficits, resulted in a loss of function, as evaluated by the MSTS scoring system, and QOL, as assessed by the EQ5D-VAS, after surgery for 247 soft tissue tumors [4]. That study also identified tumor size, bone resection, baseline MSTS, and age as confounders in a multivariate model. Similarly, Davis et al. showed that wound complications after surgery for soft tissue tumors resulted in loss of function [6] and identified tumor size and bone resection as confounding factors. These studies suggest that it is important to consider the influence of confounding factors when evaluating postoperative limb function.

Limited data is available on the effects of infections on function in bone tumor cases [5,7,8]. The first author of this manuscript previously studied the effect of infection on limb function in 125 megaprosthesis reconstruction cases for bone tumors around the knee [5]. Similarly, Sharil et al. studied the effect of infection on limb function in 54 cases of bone tumors around the knee reconstructed using endoprosthesis [8]. Ajit Singh et al. analyzed the impact of infection on functional outcomes in endoprosthesis reconstruction cases using data from 161 patients [7]. All these studies suggest the adverse effects of infection on limb function. In this study, we reconfirmed the significant impact of infection on limb function reconstructed by tumor endoprosthesis in the largest cohort used to date from the BSTT Registry. While our previous study [5] and the study by Ajit Singh et al. noted the effect of age as a confounding factor, age was not identified as a confounding factor in this study, potentially due to the presence of stronger confounders than age. Indeed, in those two studies, pelvic location and flap administration—which were identified as strong confounders in this study—were not identified as candidate risk factors for analysis. Collecting cases with pelvic tumor location, low-grade tumors, soft tissue tumors reconstructed with a prosthesis, and flap reconstructions in bone tumors seems difficult due to their scarcity. Their collection only seems possible using big data, such as that from nationwide registries. In this study, we consider that the accumulation of a large number of cases allowed us to eliminate confounding factors.

The BSTT Registry does not provide QOL evaluation results, such as TESS or SF-36. The contents of the MSTS scoring system, such as pain, activity restriction, gait pattern, and need for support, suggest that lower MSTS scores might have several effects on QOL. In addition, several studies suggested functional loss was a significant risk factor for lower QOL [10]. These findings indirectly suggest that infection might affect QOL. Indeed, limited data has been reported on the direct effects of infection on QOL [7]. Therefore, future studies should examine the direct effects of infection on QOL based on patient-oriented evaluations with a larger number of patient groups.

In this study, bone origin, pelvic bone location, high-grade histological findings, and myocutaneous flap reconstruction were used for adjustment in PSM. In previous studies, the need for plastic reconstruction [9], a high grade [6], and bone origin [11] were reported as adversely affecting QOL but not function. These findings strongly suggest that adjusting for these factors is reasonable in this cohort to effectively exclude confounders.

As shown in previous studies, while the BSTT Registry can survey several kinds of risks for infection, it lacks other significant factors critical for establishing infection, such as BMI, comorbidity, hemoglobin, albumin, blood loss, surgery duration, administration status of prophylactic antibiotics, application of intensive care unit control, and blood transfusion, some of which are considered as probable confounders [1]. Their omission is an unavoidable limitation of this study. In addition, since some analyses have shown that the preoperative MSTS score is a determinant of the postoperative MSTS score, its collection would be desirable [4]. However, the fact that it is not included in the data items of this registry is also considered a limitation. Nevertheless, the analysis remains valid and meaningful, as it was based on a sufficiently large data set that allowed for rigorous adjustment using PSM, thereby minimizing the impact of unmeasured confounders to the fullest possible extent.

The first author has previously shown that there is no difference in function among the intervention modalities for infection, such as conservative therapy, DAIR, and two-stage revision, based on a limited number of patients [5]. However, in conventional arthroplasty, better patient-reported outcome measures were reported with one-stage revision compared to two-stage revision [14]. This study did not examine differences in functional outcomes among the intervention modalities, which is another limitation of this study that should be addressed in the future.

In this study, we confirmed that the incidence of infection in soft tissue tumor cases is considerably lower compared to previous studies. Earlier reports have described infection rates in soft tissue sarcomas ranging from 9.0% to 37.1%, whereas in our cohort, the rate was only 3.4% [15,16,17,18,19]. This difference is likely attributable to the lower rate of radiotherapy application in the present cohort. Radiotherapy, a well-known risk factor for postoperative infection, was applied in 22.3% to 69.5% of cases in previous studies [15,16,17,18,19], while the rate was only 13.8% in our study. This reflects a common trend in Japanese hospitals, where radiotherapy is often avoided in cases with adequate surgical margins due to a longstanding emphasis on securing wide margins, despite this approach not being the international standard [20,21]. This regional practice should be taken into account when interpreting the present findings.

## 5. Conclusions

We reconfirmed the adverse effects of postoperative infections on lower limb function after surgery for malignant bone and soft tissue tumors using PSM on cases obtained from the BSTT Registry. While it has several limitations, the BSTT Registry is useful for functionally evaluating limbs with infections.

## Figures and Tables

**Table 1:** (**A**) Patients’ characteristics with soft tissue tumors (crude). (**B**) Patients’ characteristics with bone tumors (crude). (**C**) Patients’ pathological diagnoses.

(A)						
	Overall	(%)	No Infection	(%)	Infection	(%)
N	1099		1061		38	
Age (years) ^1^	59.2 ± 18.1		59.0 ± 18.2		64.3 ± 16.3	
Sex						
Male	570	51.9	546	51.5	24	63.1
Female	529	48.1	515	48.5	14	36.8
Tumor site						
Thigh	727	66.1	707	66.6	20	52.6
Lower leg	162	14.7	155	14.6	7	18.4
Knee	80	7.3	76	7.2	4	10.5
Buttock	63	5.7	60	5.7	3	7.9
Foot and ankle	38	3.4	36	3.4	2	5.3
Inguinal region	29	2.6	27	2.5	2	5.3
Tumor diameter (cm) ^1^	11.3 ± 9.2		11.2 ± 9.2		12.6 ± 9.2	
Tumor grade						
Low	446	40.6	443	41.7	3	7.9
High	653	59.4	618	58.2	35	92.1
Myocutaneous flap	143	13.0	135	12.7	8	21.0
Reconstruction by prosthesis	19	1.7	16	1.5	3	7.9
Chemotherapy	299	27.2	286	27.0	13	34.2
Radiotherapy	152	13.8	140	13.2	12	31.5
Follow-up (months) ^1^	42.5 ± 30.6		42.6 ± 30.7		40.2 ± 28.5	
(**B**)						
	**Overall**	**(%)**	**No Infection**	**(%)**	**Infection**	**(%)**
N	410		367		43	
Age (years) ^1^	33.8 ± 21.2		33.7 ± 21.3		34.5 ± 21.0	
Sex						
Male	235	57.3	209	56.9	26	60.0
Female	175	42.7	158	43.1	17	40.0
Tumor site						
Femur	239	58.3	221	60.2	18	41.9
Tibia	103	25.1	91	24.8	12	27.9
Pelvis	43	10.5	32	8.7	11	25.5
Fibula	16	3.9	15	4.1	1	2.3
Foot and ankle	9	2.2	8	2.2	1	2.3
Tumor diameter (cm) ^1^	10.5 ± 11.6		10.6 ± 12.1		9.7 ± 3.9	
Tumor grade						
Low	91	22.2	85	23.1	6	14.0
High	319	77.8	282	76.8	37	86.0
Myocutaneous flap	43	10.4	34	9.3	9	20.9
Reconstruction by prosthesis	215	52.4	193	52.6	22	51.2
Chemotherapy	271	66.1	240	65.4	31	72.1
Radiotherapy	13	3.2	11	3.0	2	4.7
Follow-up (months) ^1^	49.2 ± 35.5		48.0 ± 34.3		59.8 ±43.4	
(**C**)						
**Bone**			**Crude**	**(%)**	**After PSM**	**(%)**
Osteosarcoma			245	59.8	49	61.3
Chondrosarcoma			82	20.0	15	18.8
Ewing sarcoma/PNET			22	5.4	5	6.3
Undifferentiated pleomorphic sarcoma			14	3.4	3	3.8
Leiomyosarcoma			10	2.4	1	1.3
Dedifferentiated chondrosarcoma			7	1.7	1	1.3
Clear cell chondrosaorcoma			7	1.7	0	0
Others			23	5.6	6	7.5
**Soft tissue**						
Liposarcoma			487	44.3	23	30.2
Undifferentiated pleomorphic sarcoma			173	15.7	18	23.7
Myxofibrosarcoma			121	11.0	8	10.5
Leiomyosarcoma			66	6.0	6	7.9
Synovial sarcoma			46	4.2	4	5.3
Malignant peripheral nerve sheath tumor			26	2.4	0	0
Dermatofibrosarcoma protuberance			11	1.0	0	0
Extraskeletal osteosarcoma			11	1.0	0	0
Fibrosarcoma			11	1.0	0	0
Solitary fibrous tumor			10	0.9	0	0
Extraskeletal Ewing sarcoma/PNET			10	0.9	0	0
Rhabdomyosarcoma			10	0.9	2	2.6
Low-grade fibromyxoid sarcoma			9	0.8	0	0
Extraskeletal chondrosarcoma			8	0.7	1	1.3
Alveolar soft-part sarcoma			8	0.7	0	0
Epithelioid sarcoma			8	0.7	2	2.6
Clear-cell sarcoma			7	0.6	1	1.3
Others			77	7.0	11	14.4

^1^ mean ± standard deviation. Note: PNET, primitive neuroectodermal tumor; PSM, propensity score matching.

**Table 2:** (**A**) Result of multivariate analysis prior to propensity score matching in soft tissue tumors. (**B**) Result of multivariate analysis prior to propensity score matching in bone tumors.

(A)					
Variable	Level 1	Level 2	Odds Ratio	95% CI	*p*
Age (years)			0.986 ^1^	0.964–1.008 ^1^	0.22
Sex	Male	Female	0.7	0.3–1.3	0.24
Tumor diameter (cm)			0.981 ^1^	0.953–1.011 ^1^	0.21
Tumor grade	Low	High	7.6	2.2–26.8	0.002
Myocutaneous flap	No	Yes	1.5	0.6–3.3	0.37
Reconstruction by prosthesis	No	Yes	3.8	1.01–14.7	0.049
Chemotherapy	No	Yes	0.9	0.4–2.0	0.81
Radiotherapy	No	Yes	1.8	0.9–3.8	0.12
Follow-up period (months)			0.999 ^1^	0.988–1.010 ^1^	0.85
(**B**)					
**Variable**	**Level 1**	**Level 2**	**Odds Ratio**	**95% CI**	***p***
Age (years)			0.988 ^1^	0.970–1.008 ^1^	0.24
Sex	Male	Female	0.9	0.4–1.7	0.68
Tumor diameter (cm)			1.018 ^1^	0.984–1.092 ^1^	0.41
Location	Others	Pelvic bone	4.3	1.8–10.5	0.001
Tumor grade	Low	High	1.6	0.5–5.1	0.42
Myocutaneous flap	No	Yes	2.6	1.1–6.1	0.03
Reconstruction by prosthesis	No	Yes	1.1	0.5–2.3	0.74
Chemotherapy	No	Yes	1.7	0.6–5.0	0.32
Radiotherapy	No	Yes	1.0	0.2–5.4	0.98
Follow-up period (months)			0.990	0.982–0.999 ^1^	0.04

^1^ per unit. Note: CI, confidence interval.

**Table 3:** (**A**) Patients’ characteristics after propensity score matching in soft tissue tumors. (**B**) Patients’ characteristics after propensity score matching in bone tumors.

(A)						
	Overall	(%)	No Infection	(%)	Infection	(%)
N	76		38		38	
Age (years) ^1^	61.0 ± 18.8		57.6 ± 20.6		64.3 ± 16.3	
Sex						
Male	45	59.2	21	55.2	24	63.2
Female	31	40.8	17	44.7	14	36.8
Tumor site						
Thigh	43	56.6	23	60.5	20	52.6
Lower leg	17	22.3	10	26.3	7	18.4
Knee	6	7.9	2	5.3	4	10.5
Buttock	5	6.6	2	5.3	3	7.9
Foot and ankle	2	2.6	0	0	2	5.3
Inguinal region	3	3.9	1	2.6	2	5.3
Tumor diameter (cm) ^1^	11.3 ± 7.7		10.1 ± 5.7		12.6 ± 9.2	
Tumor grade						
Low	6	7.9	3	7.9	3	7.9
High	70	92.1	35	92.1	35	92.1
Myocutaneous flap	14	18.4	6	15.8	8	21.1
Reconstruction by prosthesis	6	7.9	3	7.9	3	7.9
Chemotherapy	32	42.1	19	50.0	13	34.2
Radiotherapy	19	25.0	7	18.4	12	31.6
Follow-up period (months)	38.7 ± 27.7		37.2 ± 27.1		40.2 ± 28.5	
(**B**)						
	**Overall**	**(%)**	**No Infection**	**(%)**	**Infection**	**(%)**
N	80		40		40	
Age (years) ^1^	31.1 ± 20.4		27.3 ± 18.7		34.8 ±21.5	
Sex						
Male	50	62.5	25	62.5	25	62.5
Female	30	37.5	15	37.5	15	37.5
Tumor site						
Femur	35	43.8	17	42.5	18	45.0
Tibia	23	28.8	11	27.5	12	30.0
Pelvis	17	21.3	9	22.5	8	20.0
Fibula	2	2.5	1	2.5	1	2.5
Foot and ankle	3	3.8	2	5.0	1	2.5
Tumor diameter (cm) ^1^	10.5 ± 9.8		11.4 ± 13.3		9.6 ± 4.0	
Tumor grade						
Low	12	15.0	6	15.0	6	15.0
High	68	85.0	34	85.0	34	85.0
Myocutaneous flap	12	15.0	5	12.5	7	17.5
Reconstruction by prosthesis	41	51.3	20	50.0	21	52.5
Chemotherapy	57	71.3	28	70.0	29	72.5
Radiotherapy	7	8.8	5	12.5	2	5.0
Follow-up period (months)	60.0 ± 42.5		60.9 ± 42.8		59.1 ± 42.7	

^1^ mean ± standard deviation.

**Table 4:** (**A**) Comparison of mean MSTS total scores and subscores between cases with and without postoperative infections in soft tissue tumors. (**B**) Comparison of mean MSTS total scores and subscores between cases with and without postoperative infections in bone tumors.

(A)								
	Crude				After PSM			
**Items**	No Infection	Infection	Difference (95% CI)	*p*	No Infection	Infection	Difference (95% CI)	*p*
Total	26.3	21.0	−5.3 (−7.1, −3.4)	<0.0001	23.2	21.0	−2.2 (−5.9, 1.4)	0.23
Pain	4.7	4.3	−0.3 (−0.6, −0.1)	0.01	4.6	4.3	−3 (−0.7, 0.24)	0.31
Function	4.3	3.3	−1.0 (−1.4, −0.7)	<0.0001	3.7	3.3	−0.4 (−1.2, 0.3)	0.25
Emotional	4.2	3.3	−0.8 (−1.3, −0.5)	<0.0001	3.6	3.3	−0.3 (−0.9, 0.4)	0.46
Support	4.6	3.4	−1.0 (−1.4, −06)	<0.0001	3.8	3.4	−0.4 (−1.2, 0.5)	0.36
Walking	4.4	3.4	−1.0 (−1.3, −0.6)	<0.0001	3.9	3.4	−0.5 (−1.1, 0.3)	0.19
Gait	4.4	3.3	−1.0 (−1.4, −0.7)	<0.0001	3.7	3.3	−0.3 (−1.1, 0.4)	0.40
Pain	26.3	21.0	−5.3 (−7.1, −3.4)	<0.0001	23.2	21.0	−2.2 (−5.9, 1.4)	0.23
(**B**)								
	**Crude**				**After PSM**			
**Items**	**No Infection**	**Infection**	**Difference** **(95% CI)**	***p***	**No Infection**	**Infection**	**Difference** **(95% CI)**	***p***
Total	22.4	17.0	−5.4 (−7.4, −3.4)	<0.0001	20.3	17.5	−2.8 (−6.1, 0.5)	0.09
Pain	4.6	4.3	−0.3 (−0.6, −0.04)	0.02	4.4	4.3	−0.1 (−0.5, 0.4)	0.85
Function	3.3	2.3	−1.0 (−1.5, −0.6)	<0.0001	3.1	2.4	−0.7 (−1.3, −0.03)	0.04
Emotional	3.7	2.7	−1.0 (−1.4, −0.5)	<0.0001	3.5	2.7	−0.8 (−1.5, −0.1)	0.02
Support	3.5	2.4	−1.1 (−1.7, −0.6)	0.0001	3.2	2.6	−0.6 (−1.4, 0.3)	0.22
Walking	3.9	3.0	−0.9 (−1.2, −0.5)	<0.0001	3.5	3.0	−0.4 (−1.1, 0.2)	0.18
Gait	3.5	2.4	−1.1 (−1.6, −0.7)	<0.0001	3.1	2.5	−0.6 (−1.3, 0.1)	0.09
Pain	22.4	17.0	−5.4 (−7.4, −3.4)	<0.0001	20.3	17.5	−2.8 (−6.1, 0.5)	0.09

Note: PSM, propensity score matching; CI, confidence interval.

## Data Availability

The data used in this study were obtained from the Bone and Soft Tissue Tumor Registry of the Japanese Orthopaedic Association. The registry is, in principle, accessible only to researchers who have applied for its use. As there is currently no established policy regarding public access by external parties, the data cannot be made publicly available.

## References

[1] Morii T., Ogura K., Sato K., Kawai A. (2025). Infection of surgery for bone and soft tissue sarcoma with biological reconstruction: Data from the Japanese nationwide bone tumor registry. J. Orthop. Sci..

[2] Skibber J.M., Lotze M.T., Seipp C.A., Salcedo R., Rosenberg S.A. (1987). Limb-sparing surgery for soft tissue sarcomas: Wound related morbidity in patients undergoing wide local excision. Surgery.

[3] Wilke B.K., Buckner J., Huayllani M.T., Spaulding A.C., Murray P.M., Forte A.J. (2021). Cost variance in patients with soft tissue sarcoma who develop postoperative wound complications. J. Am. Acad. Orthop. Surg. Glob. Res. Rev..

[4] Davidge K.M., Wunder J., Tomlinson G., Wong R., Lipa J., Davis A.M. (2010). Function and health status outcomes following soft tissue reconstruction for limb preservation in extremity soft tissue sarcoma. Ann. Surg. Oncol..

[5] Morii T., Morioka H., Ueda T., Araki N., Hashimoto N., Kawai A., Takeuchi K., Anazawa U., Mochizuki K., Ichimura S. (2013). Functional analysis of cases of tumor endoprostheses with deep infection around the knee: A multi institutional study by the Japanese Musculoskeletal Oncology Group (JMOG). J. Orthop. Sci..

[6] Davis A.M., Sennik S., Griffin A.M., Wunder J.S., O’Sullivan B., Catton C.N., Bell R.S. (2000). Predictors of functional outcomes following limb salvage surgery for lower-extremity soft tissue sarcoma. J. Surg. Oncol..

[7] Ajit Singh V., Balakrishnan S.D., Dhanoa A., Santharalinggam R.D., Yasin N.F. (2022). Functional outcome of infected endoprosthesis: A 20-year retrospective analysis. J. Orthop. Surg..

[8] Sharil A., Nawaz A., Nor Azman M., Zulmi W., Faisham W. (2013). Early functional outcome of resection and endoprosthesis replacement for primary tumor around the knee. Malays. Orthop. J..

[9] Kask G., Repo J.P., Tukiainen E.J., Blomqvist C., Barner Rasmussen I. (2021). Soft tissue sarcoma of lower extremity: Functional outcome and quality of life. Ann. Surg. Oncol..

[10] Saebye C., Fugloe H.M., Nymark T., Safwat A., Petersen M.M., Baad Hansen T., Krarup-Hansen A., Keller J. (2017). Factors associated with reduced functional outcome and quality of life in patients having limb-sparing surgery for soft tissue sarcomas—A national multicenter study of 128 patients. Acta Oncol..

[11] Heaver C., Isaacson A., Gregory J.J., Cribb G., Cool P. (2016). Patient factors affecting the Toronto extremity salvage score following limb salvage surgery for bone and soft tissue tumors. J. Surg. Oncol..

[12] Boyle K.K., Rachala S., Nodzo S.R. (2018). Centers for Disease Control and Prevention 2017 Guidelines for Prevention of Surgical Site Infections: Review and Relevant Recommendations. Curr. Rev. Musculoskelet. Med..

[13] Enneking W.F., Dunham W., Gebhardt M.C., Malawar M., Pritchard D.J. (1993). A system for the functional evaluation of reconstructive procedures after surgical treatment of tumors of the musculoskeletal system. Clin. Orthop. Relat. Res..

[14] Klemt C., Tirumala V., Oganesyan R., Xiong L., van den Kieboom J., Kwon Y.M. (2021). Single-stage revision of the infected total knee arthroplasty is associated with improved functional outcomes: A propensity score-matched cohort study. J. Arthroplasty.

[15] Farhan-Alanie O.M., Ha T.T., Doonan J., Mahendra A., Gupta S. (2022). Inflammatory prognostic scoring systems are risk factors for surgical site infection following wide local excision of soft tissue sarcoma. Eur. J. Orthop. Surg. Traumatol..

[16] Houdek M.T., Griffin A.M., Ferguson P.C., Wunder J.S. (2019). Morbid obesity increases the risk of postoperative wound complications, infection, and repeat surgical procedures following upper extremity limb salvage surgery for soft tissue sarcoma. Hand.

[17] Kline A., Kamalapathy P., Bruce K., Raskin K., Schwab J., Lozano-Calderon S. (2021). Nutritional predictors of wound infection in patients with lower extremity soft tissue sarcoma. Ann. Surg. Oncol..

[18] Dadras M., Koepp P., Wagner J.M., Wallner C., Sogorski A., Lehnhardt M., Harati K., Behr B. (2020). Antibiotic prophylaxis for prevention of wound infections after soft tissue sarcoma resection: A retrospective cohort study. J. Surg. Oncol..

[19] Boyle E.A., Elliott J.A., McIntyre T.V., Barnes M.E., Donlon N.E., Umair M., Gillis A.E., Ridgway P.F. (2022). Body composition is associated with operative and oncologic outcomes in the management of retroperitoneal and trunk soft tissue sarcoma. Am. J. Surg..

[20] Morii T., Sato K., Ogura K., Kawai A. (2024). Incidence and risk of infection in malignant soft tissue tumor resection: Data from the nationwide soft tissue tumor registry. J. Orthop. Sci..

[21] Kawaguchi N., Ahmed A.R., Matsumoto S., Manabe J., Matsushita Y. (2004). The concept of curative margin in surgery for bone and soft tissue sarcoma. Clin. Orthop. Relat. Res..

